# Supplementary material for: Plants Distinguish Different Photoperiods to Independently Regulate Post-Flowering Vegetative Growth and Reproductive Growth
Source: Plants (Basel). 2025 Apr 30;14(9):1368. doi: 10.3390/plants14091368 (PMC12073985; doi:10.3390/plants14091368)
Supplement: Supplementary file 1 [file plants-14-01368-s001.zip › Supplementary Figure S2.pdf]

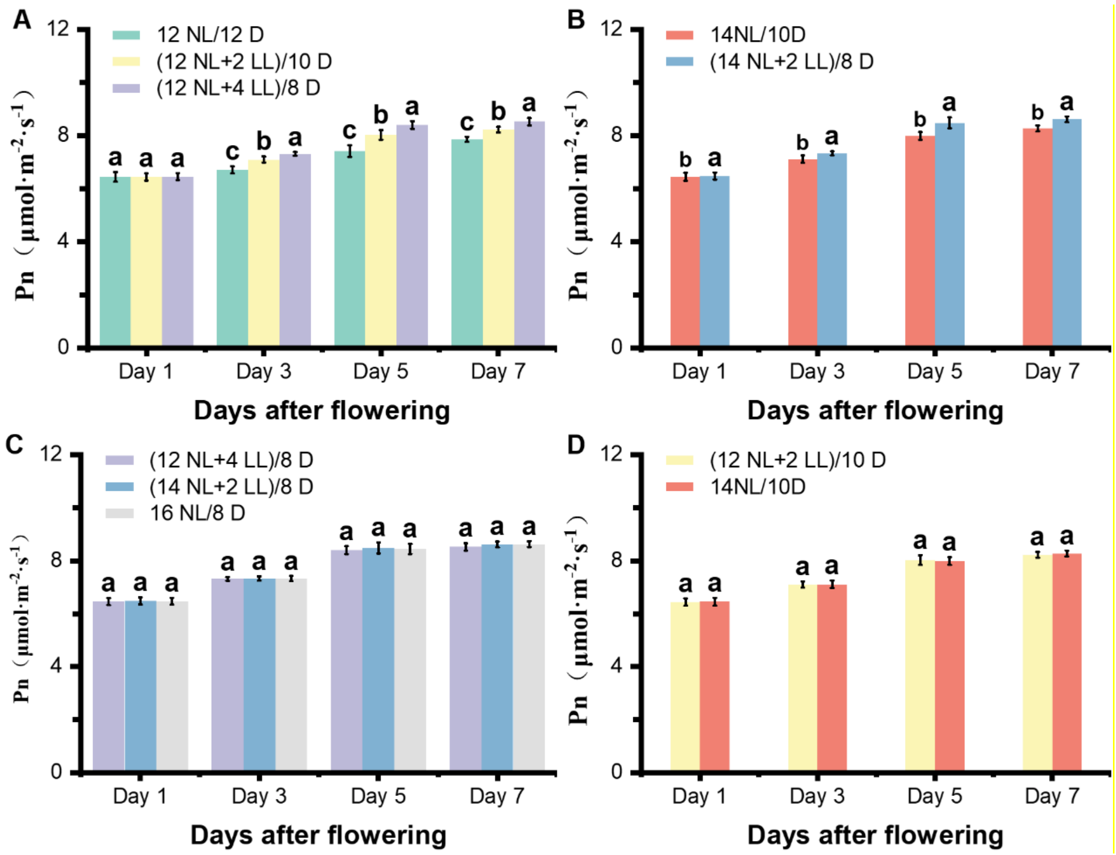

Supplementary Figure S2 Net photosynthetic rate in *Arabidopsis thaliana* rosette leaves under different post-flowering absolute photoperiod (A, B) and photosynthetic period (C, D) treatments. Photosynthetic gas exchange parameters were quantified during early vegetative development at daily intervals (1, 3, 5, and 7 days after treatment). Significant differences between treatments ($P < 0.05$) are indicated by distinct lowercase letters (one-way ANOVA with Tukey's post hoc test). Data shown as mean ± SD (n= 10 biological replicates).
