# Supplementary material for: Plants Distinguish Different Photoperiods to Independently Regulate Post-Flowering Vegetative Growth and Reproductive Growth
Source: Plants (Basel). 2025 Apr 30;14(9):1368. doi: 10.3390/plants14091368 (PMC12073985; doi:10.3390/plants14091368)
Supplement: Supplementary file 1 [file plants-14-01368-s001.zip › Supplementary Figure S3.pdf]

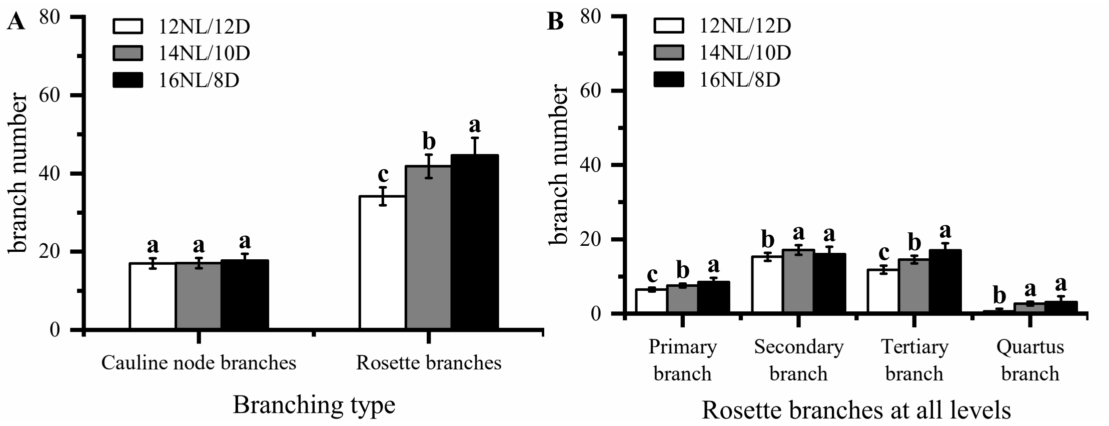

Supplementary Figure S3 Effect of normal photoperiod on the number of *Arabidopsis branches*. Distinct lowercase letters above bars denote statistically significant differences between treatments ($P < 0.05$, one-way ANOVA with Tukey's post hoc test). Values represent mean ± SD (n= 10 biological replicates).
