# Supplementary material for: Plants Distinguish Different Photoperiods to Independently Regulate Post-Flowering Vegetative Growth and Reproductive Growth
Source: Plants (Basel). 2025 Apr 30;14(9):1368. doi: 10.3390/plants14091368 (PMC12073985; doi:10.3390/plants14091368)
Supplement: Supplementary file 1 [file plants-14-01368-s001.zip › Supplementary Table S1.pdf]

Supplementary Table S1 Dry weight accumulation and partitioning patterns as affected by different post-flowering photoperiod simulations in *Arabidopsis thaliana*. Different lowercase letters within the same column indicate statistically significant differences between treatments ($P < 0.05$, one-way ANOVA, Tukey's post hoc test). Values represent mean ± SD (n = 5 plants per biological replicate, experiment consisted of three biological replicates).

Photoperiod	Group	Light/dark period	Illumination treatment	Dry weight distribution				
				Vegetative dry weight (mg)	Silique dry weight (mg)	Plant dry weight (mg)	Vegetative /plant ratio	Silique/plant ratio
Normal photoperiod	I	12L/12D	12NL/12D	233.2±6.3 c	255.2±4.2 c	488.4±9.8 c	47.7±0.5 b	52.3±0.5 a
(Experiment 1)		14L/10D	14NL/10D	299.4±4.1 b	301.4±3.0 b	600.8±6.8 b	49.8±0.2 a	50.2±0.2 b
		16L/8D	16NL/8D	329.0±4.1 a	357.0±3.8 a	686.0±7.6 a	48.0±0.2 b	52.1±0.2 a
Absolute	I	12L/12D	12NL/12D	233.2±6.3 c	255.2±4.2 b	488.4±9.8 c	47.7±0.5 c	52.3±0.5 a
photoperiod		14L/10D	(12NL+2LL)/10D	306±5.8 a	290.0±4.0 a	596.0±6.8 a	51.3±0.4 b	48.7±0.4 b
(Experiment 2)		16L/8D	(12NL+4LL)/8D	281.6±6.1 b	245.0±4.4 b	526.7±10.1 b	53.5±0.3 a	46.5±0.3 c
	II	14L/10D	14NL/10D	299.4±4.1 b	301.4±3.0 b	600.1±6.8 b	49.8±0.2 b	50.2±0.2 a
		16L/8D	(14NL+2LL)/8D	345.7±3.4 a	331.1±3.5 a	676.8±6.4 a	51.1±0.2 a	48.9±0.2 b
Photosynthetic	I	12L/12D	(12NL+4LL)/8D	281.6±6.1 c	245.0±4.4 c	526.7±10.1 b	53.5±0.3 a	46.5±0.3 c
period		14L/10D	(14NL+2LL)/8D	345.7±3.4 b	331.1±3.5 b	676.8±6.4 a	51.1±0.2 b	48.9±0.2 b
(Experiment 3)		16L/8D	16NL/8D	329±4.1 a	357.0±3.8 a	686.0±7.6 a	48.0±0.2 c	52.1±0.2 a

II	12L/12D	(12NL+2LL)/10D	306±5.8 a	290.0±4.0 b	596.0±8.3 a	51.3±0.4 a	48.7±0.4 b
	14L/10D	14NL/10D	299.4±4.1 a	301.4±3.0 a	600.8±6.8 a	49.8±0.2 b	50.2±0.2 a
